# Thyroid Dysfunction, Neurological Disorder and Immunosuppression as the Consequences of Long-term Combined Stress

**DOI:** 10.1038/s41598-018-19564-y

**Published:** 2018-03-14

**Authors:** Jingping Zhang, Jingjing Huang, Kasimujiang Aximujiang, Chenbo Xu, Abulaiti Ahemaiti, Guixia Wu, Li Zhong, Kurexi Yunusi

**Affiliations:** 1 Department of Biochemistry and Molecular Biology, Preclinical Medicine College, Xinjiang Medical University, Urumqi, 830011 China; 2 The Center of Medical Functional Experiment, Preclinical Medicine College, Xinjiang Medical University, Urumqi, 830011 China; 3 Department of Physiology, Preclinical Medicine College, Xinjiang Medical University, Urumqi, 830011 China

## Abstract

Stress is a powerful modulator of neuroendocrine, behavioral, and immunological functions. So far, the molecular mechanisms of response to stressors still remain elusive. In the current study, after 10 days of repeated chronic stress (hot-dry environment and electric foot-shock), a murine model of combined-stress (CS) was created in the SPF Wistar rats. Meanwhile, we established an ulcerative-colitis (UC) rat model induced by 2,4,6-trinitrobenzene sulfonic acid (TNBS)/ethanol enema according to previous studies. The blood, hypothalamus, and colon tissues of these rats from CS, normal control (NC), UC and sham (SH) groups, were collected for further investigations. Comparing to the NC group, the serum levels of T3, T4, fT3 and fT4 were obviously decreased in the CS group after chronic stress, indicating that thyroid dysfunction was induced by long-term combined stress. Moreover, the application of RNA-seq and subsequent analyses revealed that neurological disorder and immunosuppression were also caused in the hypothalamus and colon tissues, respectively. Comparing with SH group, besides the induced colon inflammation, thyroid dysfuntion and neurological disorder were also produced in the UC group, suggesting that hypothalamic-pituitary-thyroid (HPT) axis and gastrointestinal system might not function in isolation, but rather, have intricate crosstalks.

## Introduction

Stress is a key response pattern of animal to physical and psychological stressors, which could disturb neuroendocrine, immunological, behavioral, and metabolic functions, and adaptive physiological processes aim at reconstituting a dynamic equilibrium^1,2^. Stress response is controlled by many genetic, developmental and environmental factors^3^. Many studies revealed that stressors had the capacity to influence hypothalamic-pituitary-adrenal (HPA)^4–6^ and hypothalamic-pituitary-thyroid (HPT) axes^7,8^.

HPT axis is an intricate regulatory system. The thyroid gland can produce the thyroid hormone (TH), which is essential for the development, growth, metabolism, and necessary for the normal function of nearly all tissues, with major effects on oxygen consumption and metabolic rate^9^. Circulating TH levels are tightly controlled by a negative feedback within HPT axis, where thyrotrophin-releasing hormone (TRH) from the hypothalamus stimulates production and secretion of thyroid-stimulating hormone (TSH) by the pituitary, which in turn stimulates the synthesis, processing, and release of two forms of TH [predominant thyroxine (T4) and the active form triiodothyronine (T3)] by the thyroid gland. High TH levels signal to suppress TRH and TSH, while a drop in circulating TH will stimulate their synthesis and release. However, thyroid disorders are common in the general population, including iodine-deficient countries, and there was a 2%–8% prevalence of thyroid dysfunction according to previous studies^10^.

Thyroid dysfunction could be caused by many factors, including different kinds of stressors^8,11–13^. To gain further insight into thyroid status under different kinds of stressors, many animal models of stress have been used to assess thyroid function^8,11,13–15^. However, the relationship between the disorder of HPT axis and dysfunction of other organs was not investigated thoroughly. For example, previous study suggested that there might be closed relationships between thyroid disorders and gastrointestinal disorders^9^, but the responsible molecular mechanisms remains unclear.

This study was interested in exploring the possible cross-talks between HPT and gastrointestinal systems. Our unpublished results and previous studies indicated that the typical intense stress model complex with multiple symptoms^12^ make it difficult to explore the cross-talks between two systems. In the current study, we developed a mild combined-stress (CS) model in the SPF Wistar rats by 10 days of repeated chronic stress (hot-dry environment, electric food-shock). It has revealed that these rats of CS model suffered from thyroid dysfunction with decreased serum level of T3, T4, fT3 (free triiodothyronine) and fT4 (free thyroxine), comparing with the normal control (NC) group. Moreover, further investigations indicated that neurological disorder and immunosuppression were also induced in hypothalamus and colon respectively, implying that thyroid disorder might have effect on gut and brain^7,9,16^. In order to test whether the gastrointestinal disorders had effect on HPT axis or not, we also duplicated a TNBS-induced ulcerative-colitis (UC) rat model, according to previous studies^17,18^. The results of experiments demonstrated thyroid dysfuntion with sharply reduced serum levels of T3, T4, fT3 and fT4 in the UC group, suggesting that gastrointestinal disorders would lead to the disorders of HPT axis. Here, we report the results.

## Results

### Histological analysis for hypothalamus and colon sections

**Figure 1:**
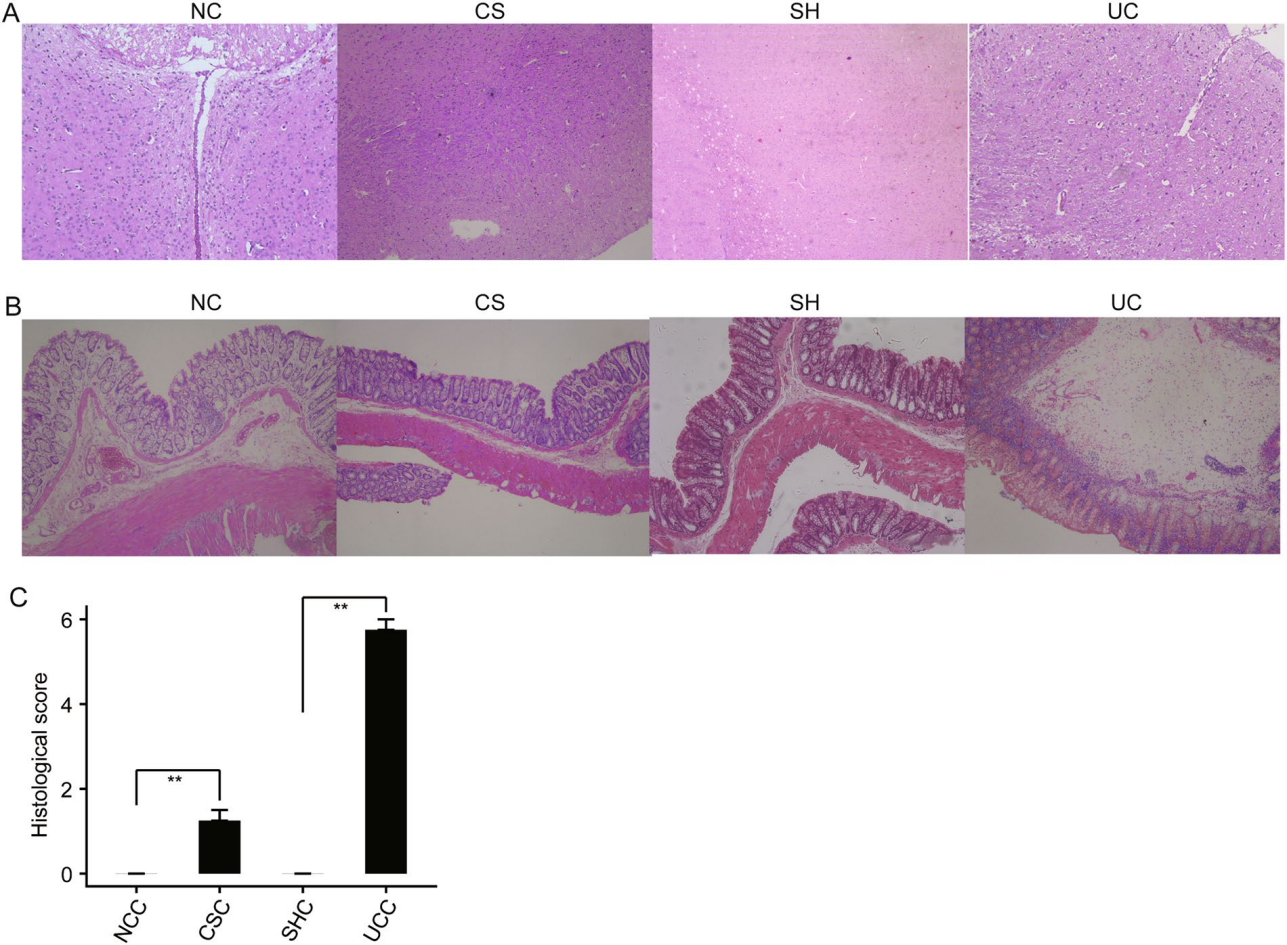
Histological analysis for hypothalamus and colon tissues of rats from normal control (NC), combined-stress (CS), sham(SH) and ulcerative-colitis (UC) groups. (**A**) Microscopic observation of hypothalamus sections from NC, CS, SH and UC groups (HE staining × 20). (**B**) Microscopic observation of colon sections from NC, CS and groups (HE staining × 10). (**C**) Colonic histological score. NCC, normal-control colon; CSC, combined-stress colon; SHC, sham colon; UCC, ulcerative-colitis colon. Results were represented as mean ± SE (n = 4). **P < 0.01, calculated with student’s *t* test.

Stress response is regulated by many parts of the brain^13^, and stress could induce damage to the colon tissue^19^. Therefore, hypothalamus and colon tissues of the rats from the NC, CS, SH and UC groups were collected to perform slice analysis. In the hypothalamus sections from CS and UC groups, cerebral vascular dilatation and congestion, widened peripheral gap could be observed (Fig. 1A), and some neurons became round, implying some damage might be induced. However, the similar morphological characters could not be found in the NC and SH groups.

A small amount of inflammatory cells presented in the mucosa and submucosal layer of colonic tissue from CS group, and there was no obvious difference in the morphological characters, comparing to the NC group (Fig. 1B). In the UC group, degeneration and necrosis of mucosal epithelial cell, and infiltration of mucosal lamina and a large number of neutrophil with cellulose exudation and hemorrhage could be observed (Fig. 1B), indicating that the serious damage was caused by TNBS/ethanol enema. These results were conformed by a quantitative assessment of the morphological manifestation (Fig. 1C). Because no damage was found in hypothalamus and colon sections from the NC and SH groups, it indicated that regular experimental operations would not cause damage to tissues in current study.

### RNA-seq data summary and Exploring Differentially Expressed Genes

Using an Illumina NextSeq. 500, we generated over 0.46 billion pair-end reads (Table S1) for 16 cDNA libraries [combine-stress colon 1 (CSC1), combine-stress hypothalamus 1 (CSH1), combine-stress colon 2 (CSC2), combine-stress hypothalamus 2 (CSH2), ulcerative-colitis colon 1 (UCC1), ulcerative-colitis hypothalamus 1 (UCH1), ulcerative-colitis colon 2 (UCC2), ulcerative-colitis hypothalamus 1 (UCH2), normal control colon 1 (NCC1), normal control colon 2 (NCC2), normal control hypothalamus 1 (NCH1) normal control hypothalamus 2 (NCH2), sham hypothalamus 1(SHH1), sham hypothalamus 2 (SHH2), sham colon 1(SHC1), and sham colon 2 (SHC2)], corresponding to an average of 28.8 million reads per sample. Using TopHat^20^, 70.1% of all reads were successfully mapped against the current rat reference genome (Rattus_norvegicus.Rnor_5.0.dna.toplevel.fa.gz) (Table S2).

**Figure 2:**
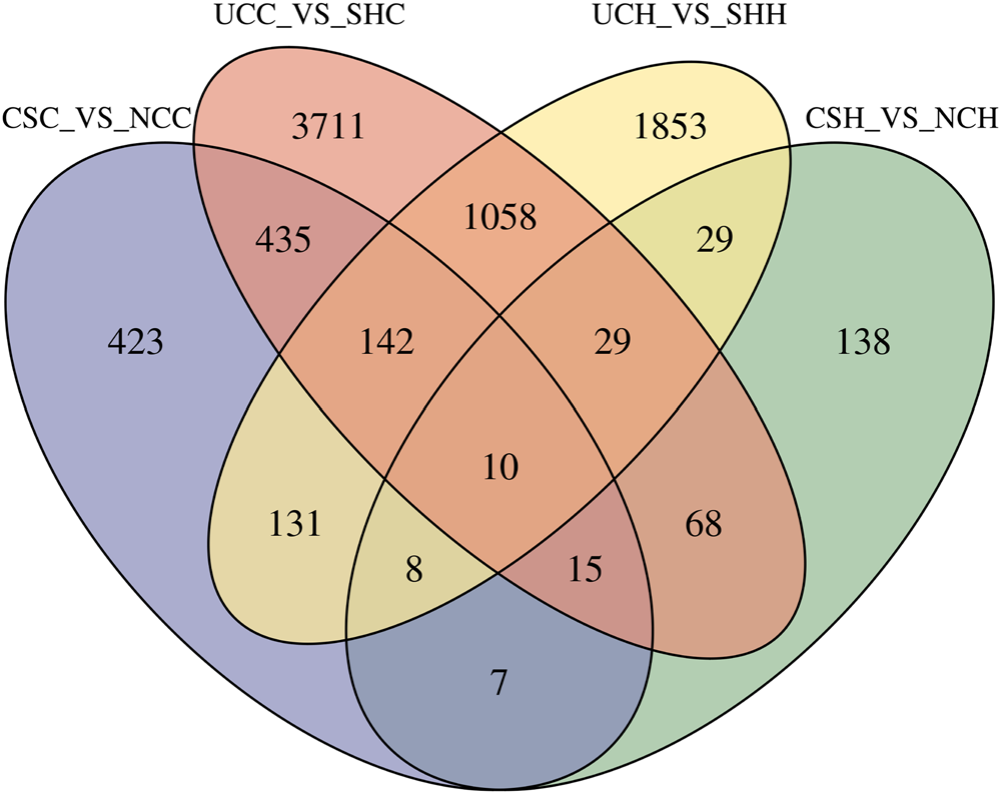
Venn diagrams of differentially expressed genes (fold change ≥ 1.5, P*-value* < 0.01) among comparisons for hypothalamus and colon tissues of rats from normal-control (NC), combined-stress (CS), sham (SH) and ulcerative-colitis (UC) groups. CSC, combined-stress colon; CSH, combined-stress hypothalamus; UCC, ulcerative-colitis colon; UCH, ulcerative-colitis hypothalamus; NCC, normal-control colon; NCH, normal-control hypothalamus. SHH, sham hypothalamus; SHC, sham colon.

Using software edge R^21^, a total of 304, 3259, 1171 and 5423 differentially expressed genes (DEGs) (FC ≥ 1.5, *P*** ≤ **0.01) were detected in the comparisons of *CSH* vs. *NCH*, *UCH* vs. *SHH*, *CSC* vs. *NCC* and *UCC* vs. *SHC* respectively (Table S3, Fig. 2).

### Thyroid dysfunction was induced in CS group

**Figure 3:**
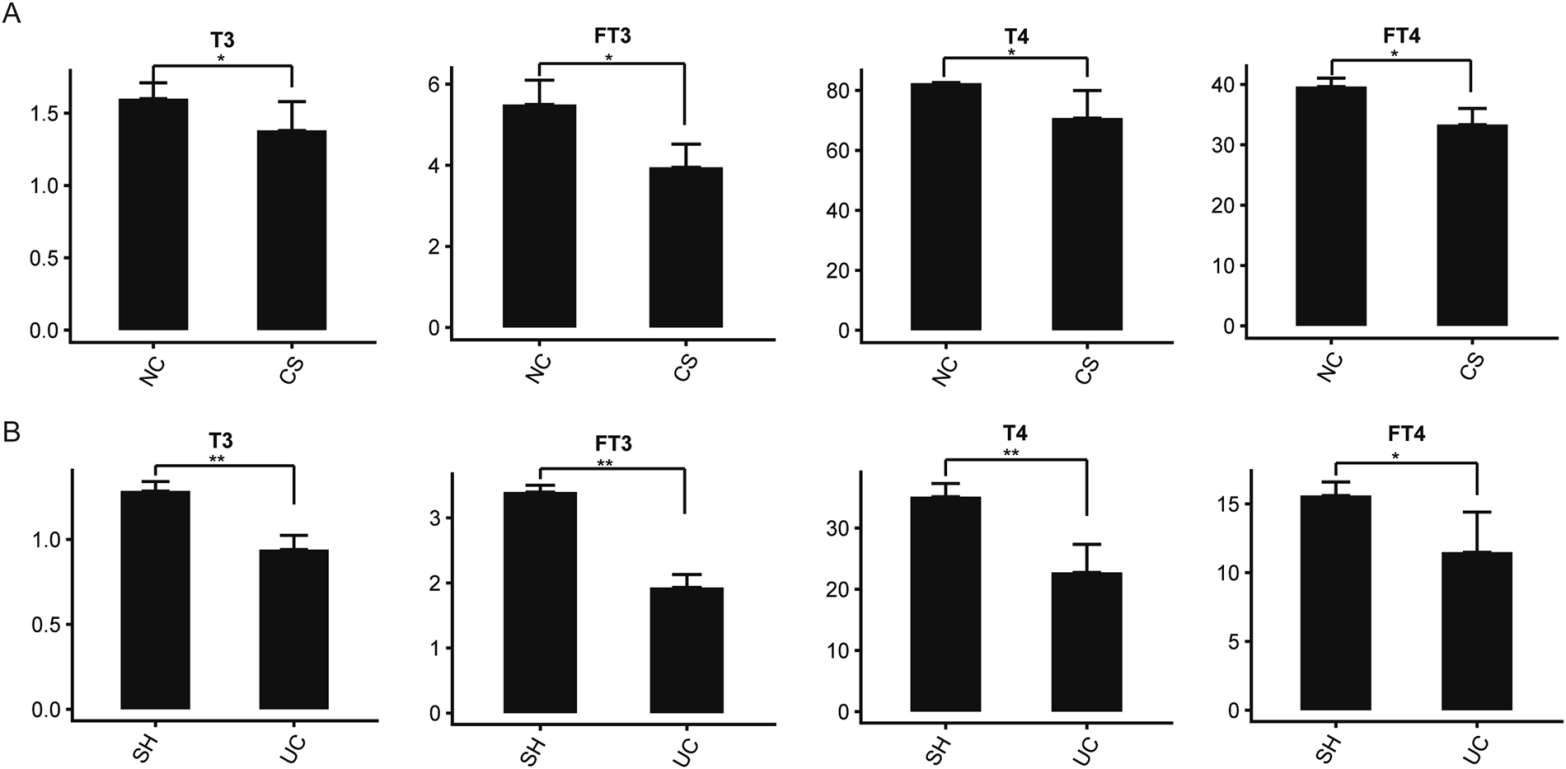
The detection for thyroid function of rats from normal-control (NC), combined-stress (CS), sham (SH) and ulcerative-colitis (UC) groups. (**A**) and (**B**) Serum levels of T3, T4, fT3 and fT4 in four groups. Results were represented as mean ± SE (n = 8). *P < 0.05, **P < 0.01, calculated with student’s *t* test.

In our former experiments, the cortisol levels and behavioral endpoints were investigated, but we found that there were no significant differences between CS and NC groups after chronic CS (data not shown). Previous study revealed that chronic stress could induce the dysfunction of thyroid^8,12^. In order to explore the thyroid function of the rats from the CS group, the serum concentrations of T3, T4, fT3 and fT4 were investigated after 10 days of repeated CS (Fig. 3A). Comparing with the NC group, it revealed that the serum levels of T3, T4, fT3 and fT4 were all reduced obviously in the CS group, indicating that thyroid dysfunction was induced by the chronic CS.

### Neurological disorder was induced in the CS group

**Figure 4:**
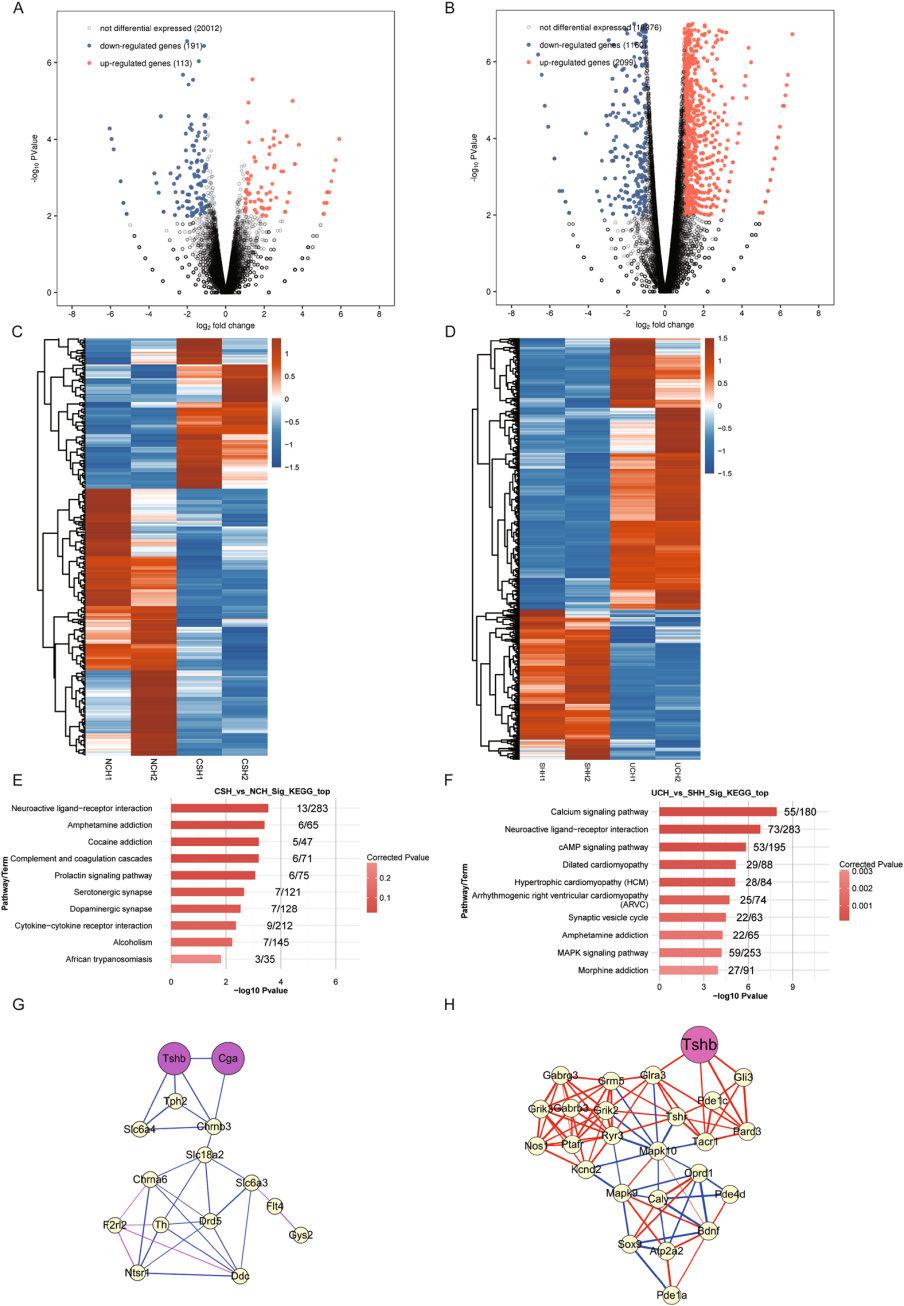
Investigations on differentially expressed genes (DEGs) for hypothalamus tissues from normal-control (NC), combined-stress (CS), sham (SH) and ulcerative-colitis (UC) groups. (**A**) and (**B**) Volcano plots indicate gene expression in *CSH vs. NCH* and *UCH vs. SHH*, respectively. The x-axis represents the log_2_ fold of changes (FC), while the y-axis represents the −log_10_ significant of difference (P-value).Genes with significant differential expression were shown in red (up-regulated gene) or blue (down-regulated gene), and genes that were not significantly differentially expressed were shown in black. A FC of log_2_ration ≥ 1.5 and P*-*value < 0.01 were set as threshold to determine the genes undergone differential expression. (**C**) and (**D**) The heat-maps for DEGs from *CSH vs. NCH* and *UCH vs. SHH*, respectively. (**E**) and (**F**) KEGG analyses of DEGs from *CSH vs. NCH* and *UCH vs. SHH*, respectively. Only the top 10 terms are listed here. (**G**) and (**H**) The network analyses under the Pearson correlation coefficients (PCCs) of these DEGs (PPC ≥ 0.95, P < 0.05) enriched in selected KEGG terms from *CSH vs. NCH* and *UCH vs. SHH*, respectively. The nodes represent DEGs. Blue and pink lines represent positive and negative correlations, respectively. CSH, combined-stress hypothalamus; UCH, ulcerative-colitis hypothalamus; SHH, sham hypothalamus; NCH, normal-control hypothalamus.

The histological analysis above suggested that some damage might be induced in the hypothalamus tissues of rats from CS group after the combined stress (Fig. 1A), and there were 304 DEGs in *CSH vs. NCH* (Fig. 2 and Table S3). Thus, KEGG pathways analysis of these DEGs was carried out (Fig. 4A,C and E). It showed that 15 KEGG terms (P < 0.05) were identified (Table S4). As is known to all, “*neuroactive ligand-receptor interaction*” (ID: rno04080), “*serotonergic synapse*” (ID: rno04726), “*dopaminergic synapse*” (ID: rno04728), and “PI3K-Akt signaling pathway” (rno04151) terms are involved in the nervous system, which are related to stress response. The abnormalities of these functional pathways suggested that long-term CS might have induced neurological disorder of the brain in CS group.

To our surprise, we found that *CGA* (*ENSRNOG00000009269*) and *TSHB* (*ENSRNOG00000016793*), which encode the α and β subunits of TSH respectively, were DEGs and enriched in “*neuroactive ligand-receptor interaction*” term. The expression of the two genes was up-regulated in the CS group, and the expression levels were extremely low in the NC group. Generally, TSH is always produced in the pituitary gland, so the abnormal expression of *CGA* and *TSHB* in hypothalamus implied the disorder of HPT axis in the CS group.

Furthermore, the network was constructed under the Pearson correlation coefficients (PCC) of these DEGs (PPC ≥ 0.95, P < 0.05), which were enriched in the 4 KEGG terms above (Fig. 4G). It was found that the *CGA* and *TSHB* were node genes of the network, suggesting that abnormal expression of TSH in hypothalamus might be an important molecular mechanism of response to chronic stress.

### Immunosuppression was induced in the CS group

**Figure 5:**
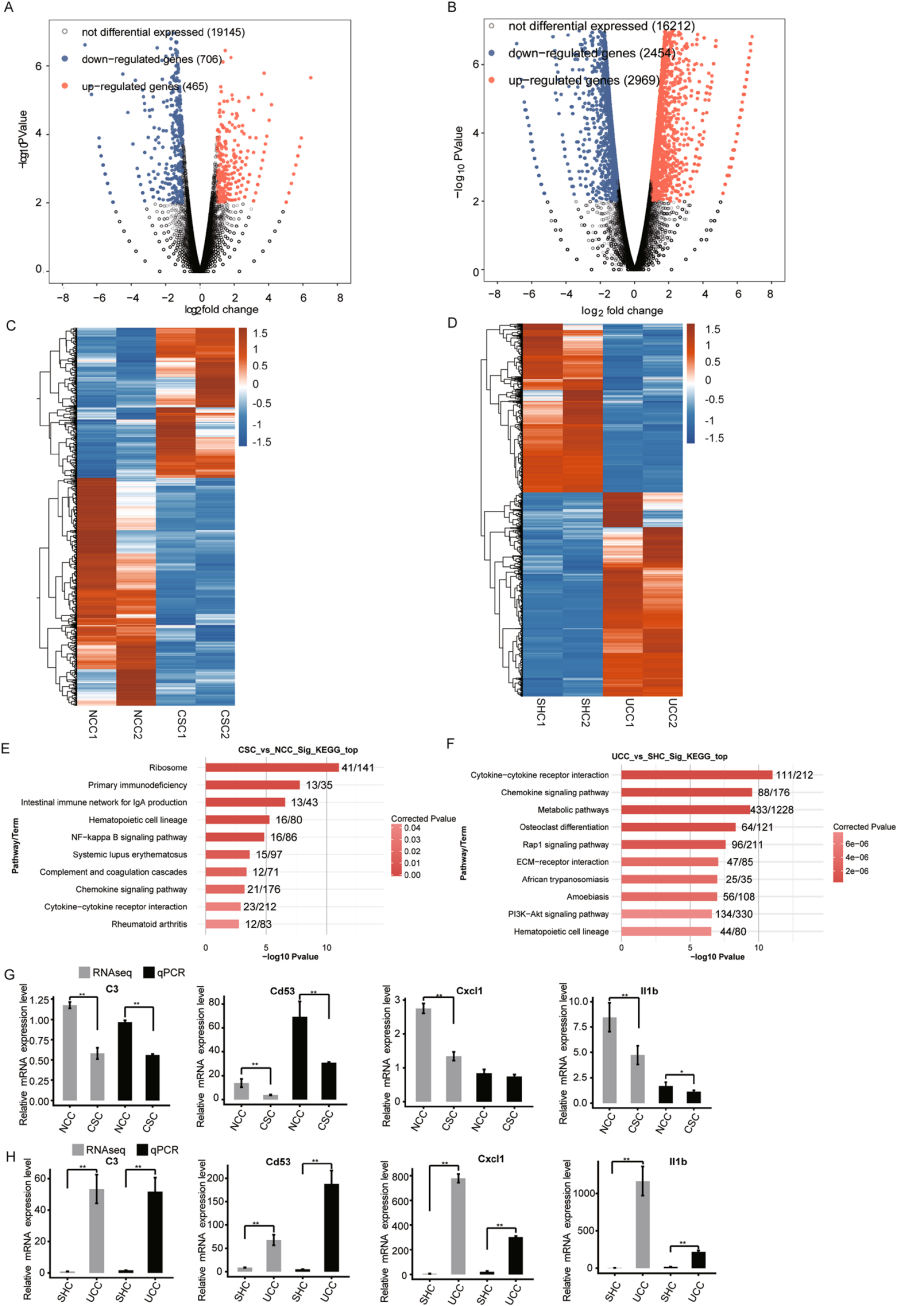
Investigations on differentially expressed genes (DEGs) in colon tissues from normal-control (NC), combined-stress (CS) and sham (SH) ulcerative-colitis (UC) groups. (**A**) and (**B**) Volcano plots indicate gene expression in *CSC vs. NCC* and *UCC vs. SHC*, respectively. The x-axis represents the log_2_ fold of changes, while the y-axis represents the -log_10_ significant of difference (P-value). Genes with significant differential expression were shown in red (up-regulated gene) or blue (down-regulated gene), and genes that were not significantly differentially expressed were shown in black. (**C**) and (**D**) The heat-maps for DEGs from *CSC vs. NCC* and *UCC vs. SHC*, respectively. (**E**) and (**F**) KEGG analyses of DEGs from *CSC vs. NCC* and *UCC vs. SHC*, respectively. Only the top 10 terms are listed here. (**G**) and (**H**) The mRNA expression levels of *C3*, *CD53*, *Cxcl1* and *IL1b* determined by high-throughput sequencing and qRT-PCR in four groups. RPKM (reads per kilobase per million mapped reads) was used to calculate the expression levels of genes in RNA-seq data, and mRNA expression levels of genes were normalized with the *GAPDH* gene CSC, combined-stress colon; UCC, ulcerative-colitis colon; SHC, sham colon; NCC, normal-control colon. Results were represented as mean ± SE. *P < 0.05; **P < 0.01, calculated with student’s *t* test.

Although histological analysis indicated that there was no obvious damage in the colonic tissue from CS group after chronic stress, there were 1171 DEGs in *CSC vs. NCC* (Table S3, Fig. 2). In order to explore this phenomenon, KEGG pathways analysis of DEGs from *CSC* vs. *NCC* was performed. It showed that 25 enriched terms were identified (P < 0.05) (Table S5, Fig. 5A,C and E). Among these terms, “*primary immunodeficiency*” (ID: rno05340), “*intestinal immune network for IgA production*” (ID: rno04672), “*complement and coagulation cascades*” (ID: rno04610), “*chemokine signaling pathway*” (ID: rno04062), “*cytokine-cytokine receptor interaction* (ID: rno04060), “*NF-kappa B signaling pathway*” (ID: rno04064), “*T cell receptor signaling pathway*” (ID: rno04660), and “*B cell receptor signaling pathway*” (ID: rno04662) terms are associated with immune system, suggesting that the chronic stress may affect the immune system of the rats from CS group indeed.

Furthermore, most of these DEGs including *CD53* (*ENSRNOG00000017874*), *Cxcl1* (*ENSRNOG00000002802*), *C3* (*ENSRNOG00000046834*) and *IL1b* (*ENSRNOG00000004649*) (Table S3), which belong to these terms associated with immune system, were down-regulated in the CS group (Fig. 5G), comparing with NC group. Immune systems are essential for animals, and the normal expression levels of cytokines and other immunity-related genes are important for health. Therefore, it could be concluded that the chronic combined stress could induce immunosuppression, with suppressing the production of proinflammatory cytokines and chemokines and downregulating the production of cytokines necessary for the generation of adaptive immune responses.

### Thyroid dysfunction was induced in the UC group

The histological analysis revealed that serious damage was caused after TNBS/ethanol enema, and a large number of DEGs were identified in *UCC vs. SHC*. KEGG analysis of the DEGs showed that 125 enriched terms were obtained (P < 0.05) (Table S6). Many functional terms were associated with immune system, including lots of DEGs which encoded cytokines and chemokines. The expression levels of these cytokines and chemokines in UC group were up-regulated comparing with the SH group (Fig. 5H), indicating that the inflammatory response was induced after TNBS/ethanol enema in the UC group.

In order to explore whether the gastrointestinal diseases could affect the HPT axis or not, the thyroid function of rats from the UC group was also detected (Fig. 3B). The results revealed that the levels of T3, T4, fT3 and fT4 were all decreased greatly in the UC group, comparing with the SH groups, indicating that serious thyroid dysfunction was induced by the UC. In addition, KEGG analysis of DEGs from *UCH* vs. *SHH* was also carried out, with 49 enriched terms being identified (P < 0.05) (Table S7; Fig. 4B,D and F). Multiple genes were enriched in “dopaminergic synapse” (ID: rno04728), “*neuroactive ligand-receptor interaction*” (ID: rno04080), “*serotonergic synapse*” (ID: rno04726), “cAMP signaling pathway” (ID:rno04024), and “calcium signaling pathway” (ID: rno04020) terms. Moreover, the network was constructed under the PCCs of these DEGs (PPC ≥ 0.95, P < 0.05), which were enriched in the 5 KEGG terms above. It indicated that *TSHB* was the node gene of the network (Fig. 4H). Hence, it suggested that UC might have the capacity to induce the disorder of HPT axis.

## Discussion

Stress is an important response pattern of animals to different kinds of stressors, which could be acute or chronic. Chronic stress has been an important factor in the development of psychopathology, for it can influence multiple physiological processes. Exposure to stressful events is able to disrupt the normal regulation of neuroendocrine axes, and most researches in this field have been mainly focused on the classic HPA axis^4^. However, recent increasing evidences indicated that HPT axis could also be in response to stress^8,12,14,22^, and thyroid disorders might be induced^11^. In the current study, after chronic stress, a CS model was created in the SPF Wistar rats. Further investigations indicated that CS model rats might suffer from declined thyroid activity without clear symptoms. Therefore, this study has provided new evidence that stress has the possibility to induce thyroid dysfunction. For the reason that the TH is necessary for the normal function of nearly all tissues, thyroid dysfunction might influence many other organs and even lead to the dysfunction of these organs^9,23–25^. In the CS model rats, the dysfunction of hypothalamus and colon was also identified, suggesting that this model might be used widely in many further studies.

Inflammatory bowel disease (IBD) is defined as a chronic intestinal inflammation, and has a high prevalence in general population. Ulcerative colitis (UC), characterized by inflammation confined to the large intestine, is one of the most common IBDs^26^. Although some studies have demonstrated an obvious increase in the prevalence of thyroid dysfunction in patients with UC compared with the prevalence in the general population, it was still uncertain whether the UC could induce thyroid dysfunction or not^27–29^. In the current study, we duplicated a TNBS-induced UC rat model according to previous studies^17,18^, and subsequent experiments revealed that thyroid dysfunction could be induced in the rats with UC. Thus, our study presented an important evidence suggesting that there might be some cross-talks between the gastrointestinal system and thyroid. Actually, both thyroid dysfunction and disorder of gastrointestinal system are common in general population, so it is necessary to explore their intricate relationship both in heath and disease, in the future.

As we know, thyroid dysfunction with decreased levels of T3, T4, fT3 and fT4 would influence the expression levels of TRH and TSH, which are produced by hypothalamus and pituitary respectively. From the RNA-seq data, although the *TRH* was not DEG both in *CSH vs. NCH* and *UCH vs. SH H*, the expression levels of *TRH* decreased and increased in CS and UC groups respectively, comparing with the NC group. Without RNA-seq data from pituitary tissue, we could not analyze the expression levels of *TSH* perfectly in the current study. But interestingly, the *CGA* and *TSHB*, which encode the α and β subunits of TSH respectively, were DEGs both in *CSH vs. NCH* and *UCH vs. SHH* (Table S3), and the expression levels of the two genes obviously increased both in CS and UC groups, comparing with the extremely low expression level in the NC and SH group. The abnormal expression of TSH in hypothalamus tissues might be induced by CS or UC in two groups, and bad for health. Hence, it could be further concluded that dysfunction of HPT axis could be induced by both CS and UC, and there might be an intricate relationship between gastrointestinal system and HPT axis.

## Materials and Methods

### Ethics statement

The approval for experiments was obtained from the Animal Ethics Committee of the First Affiliated Hospital of Xinjiang Medical University (IACUC-20121122011).

### Animal

 40 male adult SPF Wistar rats weighing 180 ± 30 g (bred at Xinjiang Medical University Animal Center, Urumqi, China) were used in the current study. These rats were housed under a thermostatically regulated condition with 12 h light/dark cycles. To ameliorate the suffering of these rats, we have made them acclimatized for one week before the initiation of the study, and have given them standard laboratory chow and water, ad libitum.

### Groups, and establishment of experimental model

A total of 40 rats were divided into four groups randomly (10 rats per group). The four groups were NC-normal control, CS- combined-stress, SH-sham, and UC- ulcerative-colitis. Five rats were kept in each cage (45 cm long × 35 cm wide × 19 cm high).

The rats from normal-control (NC) group were housed and fed in the standard conditions. The hot-dry environment was induced on the rats of combined-stress (CS) group by being kept in the climatic cabinet (Jinhong, Shanghai, China) from 11:00 am to 9:00 pm (at 26 ± 2 °C and 36%–40% relative humidity). Emotional stress was induced by the application of repeated electric foot-shocks in the electric foot-shock apparatus (20–30 V, interval 0.2–0.5 s during 20 min/day) before being housed in the climatic cabinet. The experimental routine was repeated for 10 days. This procedure was carried out in accordance with the guidelines for animal handling approved by Animal Ethics Committee of Xinjiang Medical University.

The rats from sham (SH) ulcerative-colitis (UC) groups were also housed and fed in the standard conditions. On the 10th day, rats from SH and UC group were subjected to control saline enema and TNBS/ethanol enema, respectively, under light ether anesthesia according to previous studies^17,18^. All rats were sacrificed 24 h later.

### Harvesting of blood and organ samples

On the 11^th^ day, all rats from the four groups were sacrificed by cervical dislocation under light ether anesthesia^30^. The blood, hypothalamus and colon tissues were collected quickly. Blood was harvested from abdominal aorta and collected in Vacutainer Plastic Blood Collection tubes (BD, New Jersey, USA). For each of the four groups, three rats from each cage were randomly selected and their organs were immediately frozen in liquid N_2_ and stored at −80 °C until preparation of total RNA for RNA-seq experiments. Therefore, there were two RNA-seq biological repeats for each group.

### Histological analysis and quantification

The hypothalamus and colon tissues were fixed in 4% paraformaldehyde (PFA) in PBS for 24 h and embedded in paraffin. 3-μm sections were cut using a paraffin microtome with stainless steel knives. The sections were mounted on glass slides, stained with hematoxylin-eosin (H&E) for general morphology. The stained sections were analyzed by optical microscope and digital photography integrated system (Nikon, Japan) using the affix color model.

The damage of colon sections was assessed according to a histological grading scale, which takes into consideration both inflammatory cell infiltration and tissue damage^31^. As such, the inflammatory cell infiltration was scored as follows: 0 = no infiltration; 1 = increased number of inflammatory cells in the lamina propria; 2 = inflammatory cells extending into the submucosa; and 3 = transmural inflammatory cell infiltration. The tissue damage was scored as follows: 0 = no mucosal damage; 1 = discrete epithelial lesions; 2 = erosions or focal ulcerations; and 3 = severe mucosal damage with extensive ulceration extending into the bowel wall.

### Assessment of thyroid function

Serum concentrations of T3, T4, fT3 and fT4 were measured by automatic electrochemical luminescence immunoassay system of Cobas e601 (Roche, Switzerland).

### RNA extraction and cDNA library preparation

Three whole organs from three rats in the same cage were ground together (mortar and pestle, under continuous liquid N_2_ chilling) into fine powder before RNA extraction. Ground organ tissue was stored at −80 °C. Total RNA was extracted from 30 mg ground tissue using hot phenol method. The RNA was further purified with two phenol-chloroform treatments and then treated with RQ1DNase (Promega, Madison, WI, USA) to remove DNA. The quality and quantity of the purified RNA was determined by measuring the absorbance at 260 nm/280 nm (A260/A280) using Smartspec Plus (BioRad, USA). The integrity of the RNA was further verified by 1.5% agarose gel electrophoresis.

For each sample, 10 μg of total RNA was used for RNA-seq library preparation. Polyadenylated mRNA was purified and concentrated with oligo(dT)-conjugated magnetic beads (Invitrogen, Carlsbad, CA, USA). The purified mRNA was iron fragmented at 95 °C and followed by end repair and 5′ adaptor ligation. Then reverse transcription (RT) was performed with RT primer harboring 3′ adaptor sequence and randomized hexamer. The cDNA was purified, amplified, and stored at −80 °C until used for sequencing. 12 cDNA libraries were constructed for RNA-seq.

### High-throughput sequencing

The high-throughput sequencing based on Illumina Nextseq. 500 system belongs to pair-end sequencing (ABlife Inc., Wuhan, China). The fragments generated by the cDNA libraries were sequenced from 5′ and 3′ ends to produce paired-end reads. The raw image datum obtained were transferred into nucleotide sequence datum upon base calling, generating the raw reads, and saved as ‘FASTQ’ files.

### Bioinformatic analysis

The clean reads were generated after removing adaptor sequences and low quality sequences. RPKM (reads per kilobase per million mapped reads), was used to calculate the expression levels of genes. To measure the RPKM value and screen out the differentially expressed genes (DEGs), we applied the software edge R^21^, which is specifically used to analyze the differential expression of genes using RNA-Seq data. Genes with RPKM < 0.1 in every sample were removed prior to analysis. To determine whether a gene was differentially expressed, the analysis results were based on the fold change (FC ≥ 1.5) and P-value (P ≤ 0.01).

To predict gene function and calculate the functional category distribution frequency, KEGG (Kyoto Encyclopedia of Genes and Genomes) analyses were employed using DAVID bioinformatics resources^32^. The networks were constructed by calculating the Pearson correlation coefficient (PCC) of the DEGs. Cytoscape (v3.0.2) was used to display the co-expression network^33^.

### Quantitative real-time polymerase chain reaction (qRT-PCR)

To elucidate the validity of the RNA-seq data, qRT-PCR experiments were performed for some DEGs. The relative gene expression of each gene was calculated using the Livak and Schmittgen 2^−ΔΔCt^ method^34^, normalized with the *GAPDH* gene of rat. The same RNA samples for RNA-seq were used for qPCR. In each sample, l μg of pooled RNA was reversely transcribed using the PrimeScriptTM RT Reagent Kit (Takara, Dalian, China) according to the instruction from the manufacturer. qPCR was performed on the Bio-Rad S1000 with Bestar SYBR Green RT-PCR Master Mix (DBI Bioscience, Shanghai, China). The PCR conditions are consisted of 95 °C for 10 min, followed by 40 cycles of 95 °C for 15 s, 60 °C for 1 min. Primer sequences: C3: 5′-GCG GAA GTG TTG TGA GGA TG-3′ (forward), 5′-ATG GTC TCT TCT GTG CTG CT-3′ (reverse); Cd53: 5′-CAC TGA ACT GCC AGA TTG ACA-3′ (forward), 5′-CCT TAT GGA ATG GGT GCT TTG A-3′ (reverse); Cxcl1: 5′-CGA TGG TCG TTC AAT TCC AAT T-3′ (forward), 5′-ATC TCT CCG CCC TTC TTC C-3′ (reverse); Il1b: 5′-TTT CCC TCC CTG CCT CTG A-3′ (forward), 5′-GAC AAT GCT GCC TCG TGA C-3′ (reverse); GAPDH: 5′-AAG TTC AAC GGC ACA GTC AAG-3′ (forward), 5′-ACA TAC TCA GCA CCA GCA TCA-3′ (reverse).

### Statistical analysis

The results were presented as mean ± SE. For comparison between two groups, the significance of difference between means was determined by Student’s *t-*test. A value P < 0.05 was regarded as statistically significant.

### Online data deposition

The RNA-seq data has been deposited in NCBI Gene Expression Omnibus(GEO) under accession code GSE92858.

## Electronic supplementary material

Dataset 8

Dataset 1

Dataset 2

Dataset 3

Dataset 4

Dataset 5

Dataset 6

Dataset 7
