# Supplementary material for: Thyroid Dysfunction, Neurological Disorder and Immunosuppression as the Consequences of Long-term Combined Stress
Source: Sci Rep. 2018 Mar 14;8:4552. doi: 10.1038/s41598-018-19564-y (PMC5852085; doi:10.1038/s41598-018-19564-y)
Supplement: Supplementary file 1 — Dataset 8 [file 41598_2018_19564_MOESM1_ESM.doc]

**Thyroid Dysfunction, Neurological Disorder and Immunosuppression as the Consequences of Long-term Combined Stress**

Jingping Zhang**^1^**, Jingjing Huang**^2^**, Kasimujiang Aximujiang**^1^**, Chenbo Xu**^1^**, Abulaiti Ahemaiti**^2^**, Guixia Wu**^3^**, Li Zhong**^1^** & Kurexi Yunusi**^1*^**

**^1^**Department of Biochemistry and Molecular Biology, Preclinical Medicine College, Xinjiang Medical University, Urumqi 830011, China

**^2^**The Center of Medical Functional Experiment, Preclinical Medicine College, Xinjiang Medical University, Urumqi 830011, China

**^3^**Department of Physiology, Preclinical Medicine College, Xinjiang Medical University, Urumqi 830011, China

**^*^**Corresponding author

Kurexi Yunusi, kurax8824@sina.com

**Table S1.** Statistics of RNA-seq data.

**Table S2.** Mapping of clean reads on the reference genome.

**Table S3.** The differentially expressed genes (DEGs) were detected in the comparisons of *CSH* vs. *NCH*, *UCH* vs. *SHH*, *CSC* vs. *NCC* and *UCC* vs. *SHC*.

**Table S4.** KEGG analysis of differentially expressed genes (DEGs) from *CSH* vs. *NCH*.

**Table S5.** KEGG analysis of differentially expressed genes (DEGs) from *CSC* vs. *NCC*.

**Table S6.** KEGG analysis of differentially expressed genes (DEGs) from *UCC* vs. *SHC*.

**Table S7.** KEGG analysis of differentially expressed genes (DEGs) from *UCH* vs. *SHH*.
